# Interactions at the cell membrane and pathways of internalization of nano-sized materials for nanomedicine

**DOI:** 10.3762/bjnano.11.25

**Published:** 2020-02-14

**Authors:** Valentina Francia, Daphne Montizaan, Anna Salvati

**Affiliations:** 1 Groningen Research Institute of Pharmacy, University of Groningen, 9713AV Groningen, Netherlands

**Keywords:** cell receptors, drug targeting, endocytosis, nanoparticle corona, nanoparticle uptake

## Abstract

Nano-sized materials have great potential as drug carriers for nanomedicine applications. Thanks to their size, they can exploit the cellular machinery to enter cells and be trafficked intracellularly, thus they can be used to overcome some of the cellular barriers to drug delivery. Nano-sized drug carriers of very different properties can be prepared, and their surface can be modified by the addition of targeting moieties to recognize specific cells. However, it is still difficult to understand how the material properties affect the subsequent interactions and outcomes at cellular level. As a consequence of this, designing targeted drugs remains a major challenge in drug delivery. Within this context, we discuss the current understanding of the initial steps in the interactions of nano-sized materials with cells in relation to nanomedicine applications. In particular, we focus on the difficult interplay between the initial adhesion of nano-sized materials to the cell surface, the potential recognition by cell receptors, and the subsequent mechanisms cells use to internalize them. The factors affecting these initial events are discussed. Then, we briefly describe the different pathways of endocytosis in cells and illustrate with some examples the challenges in understanding how nanomaterial properties, such as size, charge, and shape, affect the mechanisms cells use for their internalization. Technical difficulties in characterizing these mechanisms are presented. A better understanding of the first interactions of nano-sized materials with cells will help to design nanomedicines with improved targeting.

## Introduction

Nano-sized materials are widely studied in nanomedicine for their potential use as drug carriers, in imaging, and for diagnostic purposes [1–3]. Because of their size, they can interact with cells in similar ways as other nano-sized objects, such as proteins, cholesterol particles, and virus particles. These natural nano-sized objects are usually recognized by specific cell receptors at the plasma membrane and they are internalized by cells using the cell endocytic machinery [4]. Similarly, engineered nano-sized materials can exploit the cellular machinery to be internalized by cells. In fact, since the cell membrane blocks diffusion of complexes larger than ca. 1 kDa, nano-sized materials, such as nanomedicines, are transported into cells using energy-dependent mechanisms, unlike many small drugs currently present on the market [5]. This enables nanomedicines to potentially overcome problems associated with the passive diffusion of small molecular drugs through cell membranes, such as their indiscriminate internalization in different cell types and organs, which is often associated with side effects [6]. Additionally, nanomedicines can encapsulate different types of hydrophilic and hydrophobic drugs, and they can be designed to control their release profile [7]. Several other characteristics of nanomaterials such as size, material, shape, surface charge, hydrophobicity, roughness, and elasticity can be tailored in order to meet various needs [3,8]. This high engineering potential can be exploited to control the distribution and behaviour of nanomedicines in biological environments. By tuning nanomedicine design, parameters such as serum–protein interactions, sequestration by the immune system, blood circulation time, biodistribution, and cellular recognition and internalization can be tailored [1–3,7–8]. Moreover, the surface of nanomedicines can be engineered by introducing functional groups to reduce clearance and increase biodistribution, as well as for active targeting purposes [1–2,9–10]. In fact, nanomedicines can be engineered to interact with specific cell receptors, opening up new strategies for targeting specific cell types and organs [9–12]. Despite this high engineering potential, active targeting remains one of the major challenges for nanomedicine success [13–14], and so far only few targeted nanomedicines are currently present in the market, even if several are in clinical trials [6].

Recent advances in the field have shown the complexity of achieving targeted uptake by specific cells. For example, it has been shown that the biomolecules adsorbing on the nanoparticles once they are introduced in biological environments and the resulting corona can screen the targeting moieties [15–16]. At the same time, it has emerged that the corona itself can be recognized by receptors at the cell membrane [17–18] and that this initial recognition can affect the mechanism that cells use for the internalization of the nanoparticles [18]. However, several aspects of the initial recognition of nano-sized materials by cell receptors and of the molecular mechanisms leading to their uptake and intracellular processing are still unclear [19–21]. A better understanding of these processes can help to design smarter nanomedicines and to achieve better targeting [22].

Within this context, in this review we will summarise the current understanding of the very first steps of the interactions of nano-sized materials with cells, with a particular focus on the initial recognition at the cell membrane and the following mechanisms of internalization by cells. We discuss these aspects in relation to the application of nano-sized materials for nanomedicine. Challenges in characterizing these first events will be illustrated, together with a brief description of the known endocytic pathways in cells.

## Review

### 1 Interactions of nano-sized materials at the cell surface and recognition by cell receptors

#### 1.1 Active targeting

**Figure 1:**
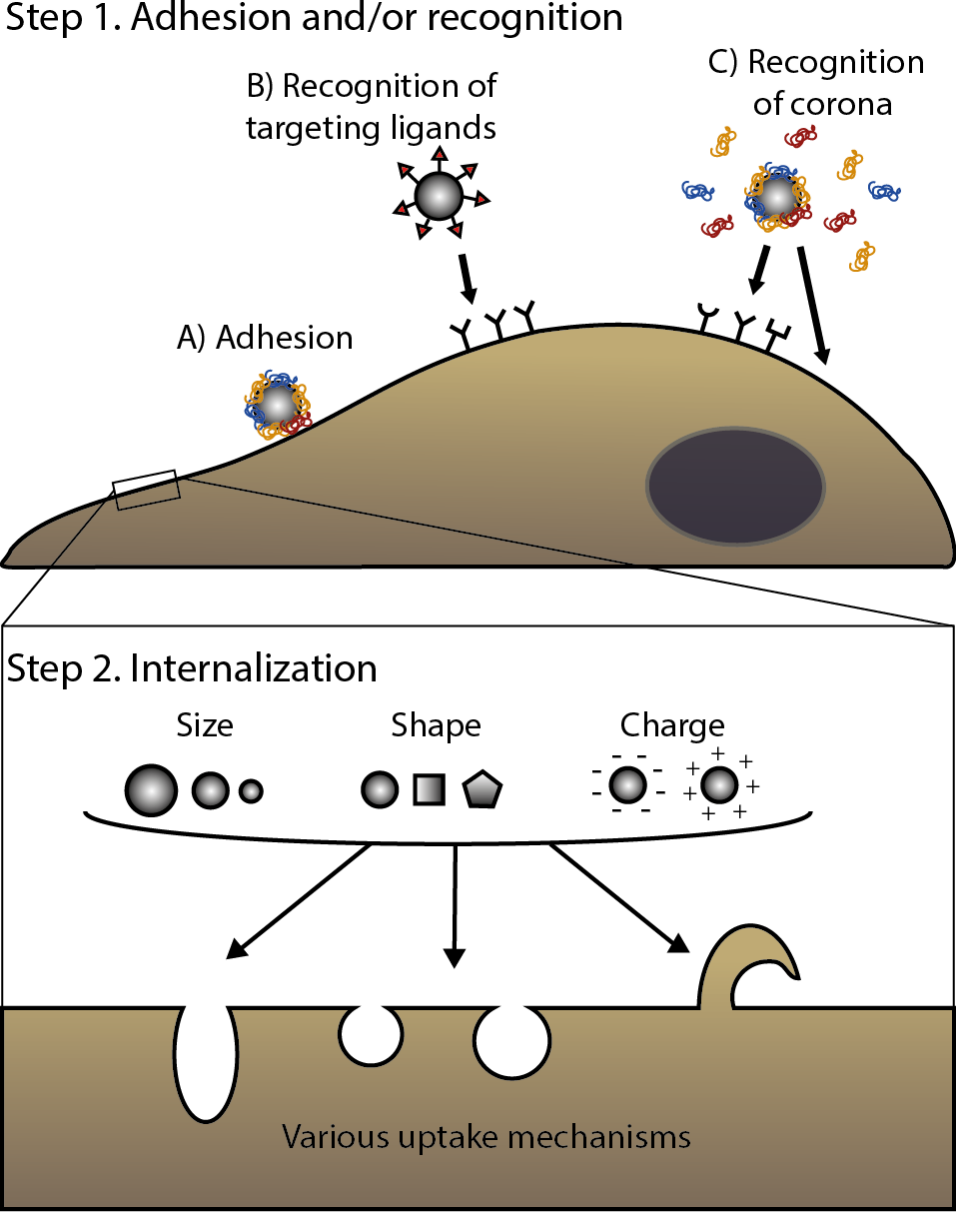
Interaction of nano-sized materials at the cell surface. First, nanoparticles adhere at the plasma membrane (A) and/or are recognized by cell receptors. Recognition can be achieved via targeting moieties, in the case of targeted nanomedicines (B) but also via the biomolecular corona (C) [10–11,17–18]. Secondly, nano-sized objects are internalized via various mechanisms (here illustrated by different shapes in the cell membrane or a membrane protrusion). However, we do not know yet how nanomaterial properties (such as size, shape, charge, as illustrated in the figure) affect or determine the mechanism cells use for the internalization [7,19–22].

The first steps in nanoparticle–cell interactions are those happening at the cell surface, including the adhesion of nanoparticles to the cell membrane and the potential interaction with cell receptors (Figure 1). In order to control and affect these first events, nano-sized carriers can be modified with targeting moieties, such as peptides, proteins, or antibodies to specifically recognize receptors on the cell surface to achieve active targeting [9–12]. These surface-functionalized nanomaterials should be internalized preferentially by cells that overexpress the targeted receptors. Examples of targeting moieties often exploited in nanomedicine are transferrin and folate, which target tumour cells overexpressing the corresponding receptors [23–24], or hyaluronic acid, which directs nanocarriers to CD44-overexpressing tumour cells [25], among many others. While many new targeted nanomedicines are developed, just few of them are currently present on the market [6]. In fact, achieving efficient targeting in vivo remains a crucial challenge for drug delivery and recently the debate on the success of nanomedicine for delivering drugs to their target has been very active [26–28].

Indeed, it is difficult to design nanoparticles that achieve specific targeting [7,9,29]. This is not only because a better understanding of the factors controlling the very first interactions of nano-sized materials with live cells is still needed (as we discuss here), but also because of challenges related to nanoparticle design and presentation of the targeting moiety. For instance, chemical coupling can affect the binding affinity of the targeting ligand to its receptor [30]. Moreover, it is difficult to control the targeting ligand density and its orientation. Both factors are important for the recognition by cell receptors and can affect cellular uptake [31–32]. Several reviews have summarized these and other similar challenges in the surface functionalization of nano-sized drug carriers to achieve targeting [33–35]. Ideally, by better controlling the early interactions with cells, nanomedicines should be recognized by the desired receptors and be trafficked intracellularly to their target.

#### 1.2 Corona formation

Another complication in achieving targeting is the formation of the biomolecular corona. When a nanomedicine (or any nano-sized material) comes in contact with a biological environment (for example, blood, interstitial fluids, or extracellular matrices) after administration, its surface is rapidly covered by various biomolecules leading to the formation of a corona [36–39]. It has been shown that, in some cases, the presence of the corona can mask the targeting moieties grafted on the nanoparticle surface, preventing recognition by cell receptors [15–16,40]. Corona formation can affect not only the targeting ability, but also particle size, stability, and overall surface properties [36]. Recent guidelines have started to highlight the importance of testing nanomedicines in the presence of relevant biological media in order to take corona effects into account [41].

Several strategies have been developed to try to reduce protein adsorption and corona formation. This can be achieved for instance by grafting hydrophilic polymers such as polyethylene glycol (PEG) on the surface of nanomedicines, or by introducing zwitterionic modifications to make nanomaterials almost neutral [42–45]. These modifications reduce the amount of biomolecules bound on the surface of nanomedicines after administration (though it has been shown that PEGylated surfaces can still adsorb proteins [46–47]) and usually also lead to decreased uptake by cells.

At the same time, the corona confers a new biological identity to nanomaterials and can affect the way nanomedicines are recognized and processed by cells [3,7–8,20–21,36,48]. Biomolecules present in the corona can, per se, have targeting capabilities towards particular receptors [17–18,49–52]. For example, apolipoprotein B and immunoglobulin G in the corona of 100 nm silica nanoparticles incubated with human serum were found to interact with their corresponding receptors, low-density lipoprotein receptor and Fc-gamma receptor I, respectively [17]. Similarly, lipid nanoparticles were efficiently targeted to the hepatocytes upon adsorption of apoE on their surface following administration [52–53]. Thus, controlling the corona composition can possibly provide new ways to control the initial interactions of nano-sized materials with cells.

The corona composition depends on nanoparticle physicochemical characteristics, such as size, shape, charge, hydrophobicity, rigidity and surface characteristics [3,7–8,48,54]. By changing these properties, the corona composition might be tuned to contain components that bind to specific cell surface receptors and initiate internalization [17,49,55–57]. Similarly, artificial coronas can be formed to achieve recognition by specific receptors. For instance, Tonigold and colleagues have shown that pre-adsorbed antibodies, which could be seen as a form of pre-formed corona, kept, at least partially, their targeting ability in the presence of serum [40].

From a broader perspective, the effects of the corona on the interactions of nanoparticles with cells are being more and more recognized [41,58–59]. For example, multiple attempts have been made in trying to predict how the presence of the corona affects targeting of nanomedicines [60–61]. Similarly, it is known that the corona composition changes not only with nanoparticle properties [3,7–8,48,54], but also depending on serum origin [62–63], serum preparation [63–65], serum concentration [18,66–67] or health status [68–69]. However, many more facets of corona effects on nanoparticle–cell interactions still need to be understood, and even more so if one aims to exploit the corona for targeting.

**Figure 2:**
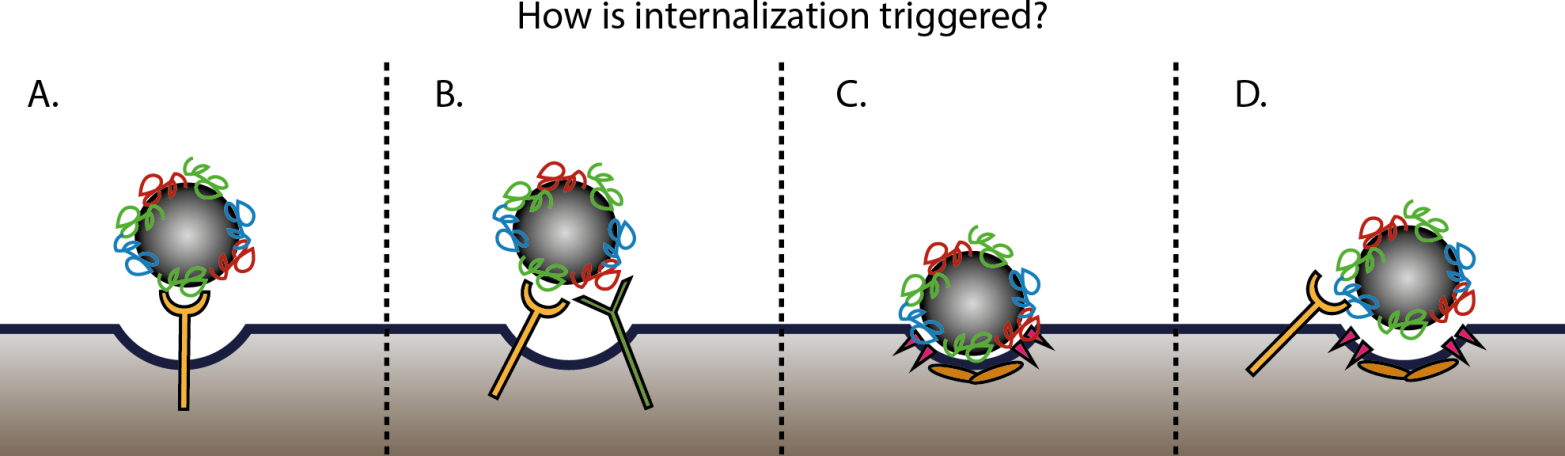
Scheme of possible scenarios that can occur at the cell surface, resulting in nanoparticle uptake. Nanoparticles or their corona can be recognized by one (A) or multiple types of receptors (B), and the recognition by receptors triggers nanoparticle uptake. Alternatively, the adhesion of the nanoparticle to the cell surface may induce internalization without receptor engagement (C) or the receptor is only involved in the initial adhesion of the nanoparticle, then uptake is triggered by different mechanisms, possibly induced by the nanoparticle itself (D).

Similarly, how receptor interactions affect the subsequent internalisation is also not known. Nano-sized materials and nanomedicines may interact only with one type of receptor (Figure 2A) or with multiple receptors at the same time (Figure 2B), and the recognition by cell receptors triggers uptake. It might be that only high-affinity interactions contribute to the entry, or that internalization occurs after receptor clustering. Alternatively, internalization may happen without recognition by specific receptors (often referred to as unspecific binding and unspecific uptake), possibly triggered by the nano-sized object itself (Figure 2C). Another possibility is that the recognition by cell receptors is involved only in the initial adhesion to the cell membrane, but not in the internalization (Figure 2D). A combination of all these different possibilities may as well be present. In all cases, addressing these open questions relative to the first interactions at the cell surface is required to understand how to achieve a more efficient targeting of nanomedicines.

### 2 Internalization

After the very first interactions at the plasma membrane, most nano-sized materials are internalized by cells [19–22]. Many questions are still open on how the initial interactions at the cell membrane affect the mechanisms cells use for the subsequent internalization. For instance, does receptor recognition trigger internalization via the same pathway used for its physiological ligands? Or do the receptors mediate just the initial adhesion and is it the nano-sized material itself that triggers its own internalization, perhaps by other ways (as also illustrated in Figure 2D)? How are uptake efficiency and the mechanisms of internalization modulated by the type of receptor engaged and/or by the initial interactions at the cell membrane? These are examples of the many questions that the field needs to address in order to control nanomedicine design to achieve the desired outcomes at cell level.

In the following sections, we will summarize key aspects of the main mechanisms of cellular internalization, i.e., endocytosis. Then, examples of works trying to understand how nanomaterial properties affect the mechanisms of uptake by cells are presented to illustrate the complexity of outcomes observed and the difficulties in drawing conclusions.

#### 2.1 Pathways of endocytosis in cells

**Figure 3:**
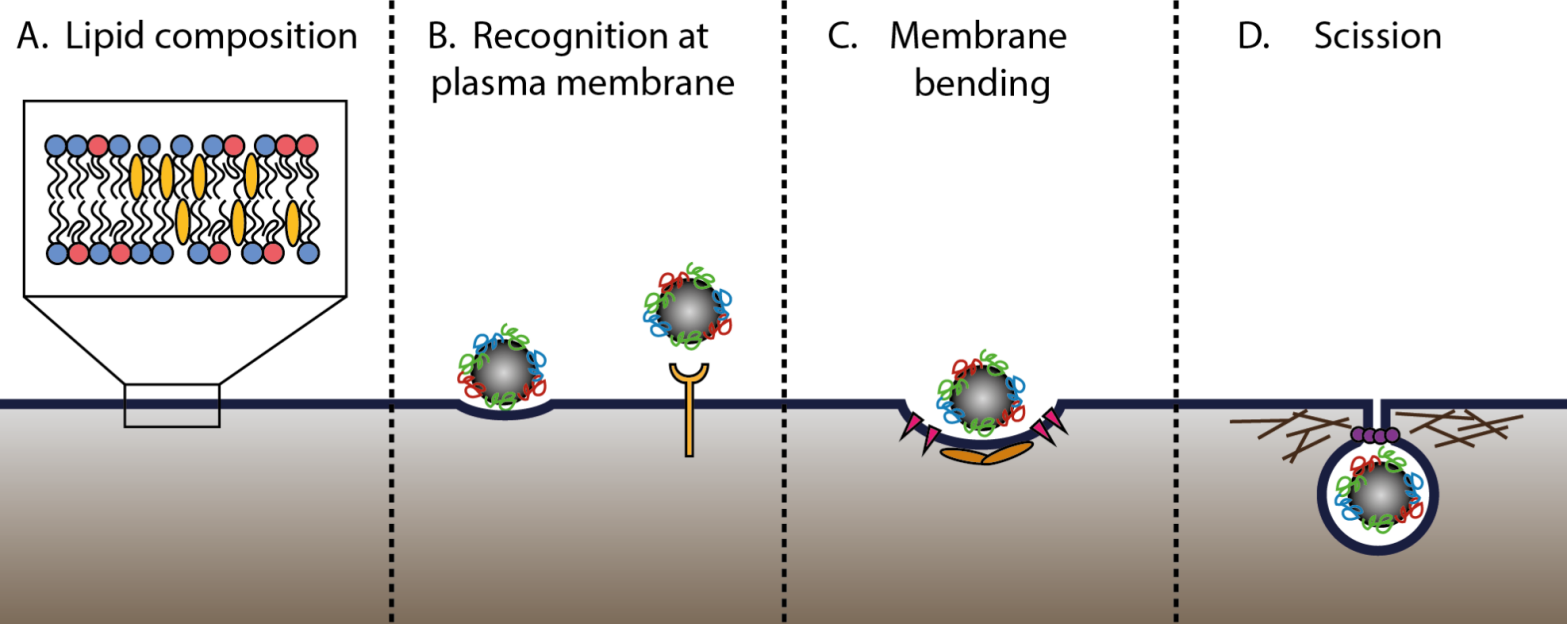
Common features of endocytic pathways [73]. Endocytosis requires a specific cell membrane composition at the site of uptake (A) [72,74–76] and starts with the recognition of the cargo at the cell membrane (B). Subsequently, membrane bending takes place to form a vesicle (C) [72–73,77–78,84]. Lastly, the vesicle is cleaved from the cell membrane via different mechanisms, which can involve various cellular components (e.g., actin and dynamin, here depicted as lines and circles, respectively) (D) [79–80].

Cells developed several mechanisms of endocytosis in order to select and sort different cargoes to their intracellular destination [70–71]. Although these mechanisms differ strongly, they also share a series of common features. As discussed by Johannes and colleagues [72–73], common features required for uptake to occur are (Figure 3):

- a specific lipid composition of the cell membrane at the site of endocytosis (such as the presence of sphingolipids or cholesterol) [70,72,74–76],
- cargo recognition at the cell membrane (receptor-mediated or not) and capture (into coated vesicles or specific carriers) [72–73],
- membrane bending, which occurs through different mechanisms, including the insertion of hydrophobic protein motifs in the membrane, local recruitment of membrane-bending domains, or scaffolding by proteins (the classic example being clathrin) [72–73,77–78], and
- scission of the endocytic vesicle, which can be guided by actin, dynamin and/or other proteins [79–83].

The specific details of each of these steps in the various endocytic mechanisms are extensively discussed in different excellent and specialized reviews [70–71], including some focused on the mechanisms of endocytosis of nanomedicines [19,21–22,85–86]. Here, we briefly summarize some of their key aspects.

One of the most studied endocytic mechanism is clathrin-mediated endocytosis (CME). CME is a form of receptor-mediated endocytosis that is used for the internalization of various biomolecules among which low density lipoprotein for cholesterol uptake and transferrin for iron uptake. After binding of the ligand to its receptor, clathrin, the main actor in CME, is recruited at the cell membrane together with several other proteins and assembles around the forming vesicle to form a clathrin-coated pit. The GTP-binding protein dynamin is then required for the scission of the clathrin-coated pit to form an endocytic vesicle. Although it was thought to be non-essential for CME [87], there are indications suggesting that actin filaments are also involved in the scission [88–89] and especially in the uptake of larger objects [90] via this pathway.

Next to CME, several clathrin-independent endocytic (CIE) pathways have been described [71,91–92]. One of these pathways is macropinocytosis, which cells use to internalize larger volumes of extracellular fluids and solutes. In macropinocytosis, extracellular fluids are engulfed by membrane ruffles and protrusions. Formation of these ruffles requires actin nucleation and in some cells also cholesterol [93–94].

Another frequently studied clathrin-independent pathway is the so-called caveolae-mediated endocytosis. Caveolae are 50–80 nm sized cup-shaped invaginations of the plasma membrane, consisting of lipid rafts enriched in cholesterol and sphingolipids, and coated with caveolins [95–97]. Endocytosis of these invaginations can be both receptor-dependent and -independent and requires actin and dynamin [96,98–99]. Nevertheless, the role of caveolae in endocytosis is currently being debated. Some suggest that caveolae are involved in transcytosis in endothelial cells [100]. According to this hypothesis, caveolae rapidly detach from the apical side of the membrane and fuse with the basal one, or directly form transient pores in thin endothelial cells [101]. Other studies have shown that in many cell types caveolae are normally not involved in endocytosis, but are stable invaginations present at the cell surface [102–103], and only undergo endocytosis upon stimulation [96,104].

Apart from these mechanisms, phagocytosis is a form of receptor-mediated endocytosis of large particles (above 0.5 µm), which requires the involvement of the cytoskeleton for membrane rearrangements. Professional phagocytic cells of the immune system use it to internalize pathogens [105]. However, it has emerged that also non-specialized phagocytic cells can internalize large particles [71,106].

Finally, other clathrin-independent endocytic mechanisms have been described. These include pathways mediated by flotillins, ADP-ribosylation factor 6, endophilins, or tubular structures called clathrin-independent carriers (CLICs). The exact machinery involved in these various clathrin-independent pathways is still investigated and the involvement of components like actin, cholesterol, or dynamin is often debated [71,91–92].

Overall, endocytosis is highly complex and still a very active field of research. This is one of the factors which makes the characterization of the mechanisms by which nano-sized materials enter cells challenging.

#### 2.2 Endocytosis of nanoparticles: effects of material properties

As we described in the Introduction, the capacity of nano-sized objects to interact with the cellular machinery has opened up the possibility of using nano-sized materials to deliver drugs to their target. Nanoparticle design can be tailored to target specific cell types or pathways. Size, charge, shape [107], hydrophobicity [108], rigidity [109–110], roughness [111] and surface functionalization [43,112] of nanomaterials are all parameters that in principle can be tuned in order to affect the pathway of internalization of nano-sized materials and ideally also to direct them towards a specific intracellular location. Still, there is not yet an agreement in the scientific community on the pathways that nano-sized materials, including nanomedicines, use to enter cells [21–22,113–114]. A better characterization of the mechanisms that cells use to internalize nano-sized materials can potentially help us to understand how to tune their design to achieve the desired outcomes at cell level [22] (as we illustrate in Figure 1).

**Nanoparticle size:** A fundamental parameter that seems to affect the pathway of internalization of nanoparticles is their size. A general observation is that the uptake efficiency of nanomaterials decreases with increasing particle size [115–117], probably because of the extensive membrane rearrangements needed for internalization of larger objects [59,118–119]. Some studies have compared explicitly the uptake efficiency of particles of different sizes trying to define the optimal size for uptake [120–123]. Additionally, it is commonly believed that most nanoparticles with sizes compatible with the size of clathrin-coated pits enter through clathrin-mediated endocytosis [115,124] and, vice versa, that larger ones do not. It was thought that the geometry and 3D structure of clathrin would not allow it. However, results opposing this general idea about size have also been reported [116,125–127]. For example, in one study using HEK293 cells, spherical polystyrene nanoparticles of 100 nm were internalized through actin-dependent but clathrin-independent processes, and vice versa, 200 nm nanoparticles entered by clathrin-mediated endocytosis [127]. Similarly, the uptake of 500 nm PRINT particles was reduced by chlorpromazine (an inhibitor of clathrin-dependent endocytosis) [125] and it has been shown that larger particles could be internalized in pits coated with plaques of clathrin [128–130].

These studies, selected just as examples among many others, already highlight the difficulties in establishing a general rule on how nanoparticle size affects uptake. Similarly, it was believed that particles larger than 200 nm could not be internalized by non-phagocytic cells [7], while opposite observations are often reported. For instance, even cubic nanoparticles of 3 μm could be internalized by HeLa cells [125].

Moreover, the effects of nanoparticle size on the mechanism of uptake may be different in different cell types. For example, it has been shown that murine RAW 264.7 macrophages have a higher uptake efficiency for carboxylated polystyrene nanoparticles compared to human endothelial HCMEC or epithelial HeLa cells [131]. In another study using carboxylated polystyrene nanoparticles of different sizes in different cell types, actin was required for the internalization of nanoparticles of 200 nm, but not for those of 40 nm in 1321N1 astrocytes. Instead, in lung epithelial A549 cells, for both nanoparticle sizes, the uptake was not dependent on actin [132]. Unfortunately, so far, only a few studies have investigated in a systematic way how different cell types internalize nanoparticles of different size, making it difficult to draw conclusions [132–134].

**Nanoparticle charge:** Apart from size, charge is another easily tunable parameter that can greatly influence the behaviour of nanoparticles in biological media [135] and on cells [136]. In general, positively charged nanoparticles seem to be internalized more efficiently than neutral and negatively charged particles [125,137–138]. However, there are other examples showing exactly the opposite [139]. It has also been reported that uptake increases with charge density (either positive or negative) [140]. Regarding the pathway of internalization, some studies suggested that positively charged nanomaterials are predominantly internalized through clathrin-mediated endocytosis, with a fraction of particles utilizing macropinocytosis, while negatively charged nanoparticles are more likely to use a cholesterol-dependent mechanism for their internalization [21,141]. Contradicting results were reported in which clathrin-mediated endocytosis did not seem to be a relevant pathway for neither positively nor negatively charged nanomaterials, while cholesterol-mediated uptake seems to be equally important for both [142]. Similarly, another study suggested that both negatively and positively charged poly(ethylene glycol)-ᴅ,ʟ-polylactic acid particles entered, at least partially, by clathrin-mediated endocytosis and macropinocytosis [143].

It is important to mention that many studies investigating the effect of charge or other nanomaterial properties on cellular interactions were performed in the absence of proteins from biological fluids such as serum. This represents a further issue since the charge of nanomaterials tends towards neutrality upon corona formation, once they are applied in a biological environment. Thus, nanoparticles that in water possess different charges might end up having all a similar charge, close to neutrality upon exposure to biological media [135]. Because of this, it is important to determine whether some of the described charge-related effects disappear once the nanomaterials are applied in a biological environment.

**Nanoparticle shape:** Another tunable parameter that can influence nanoparticle–cell interactions is shape. Simulations indicated that based on the energy required for membrane bending, the uptake would be the highest for spheres followed by cubes, then rods, and lastly discs [144]. Nevertheless, a recent in vitro study using gold particles, showed that the number of internalized particles was decreasing from rod to cubic, to spherical, to prism-shaped nanoparticles [145]. Often the effect of shape is studied by changing nanoparticle aspect ratio. Most of these studies showed that the uptake is higher when the aspect ratio is smaller [120,146–147]. This could be explained by the higher energy required to wrap a lipid membrane around a nanoparticle with high aspect ratio [148]. However, also in relation to this aspect, conflicting results were found in which cross-linked poly(ethylene glycol) hydrogels or mesoporous silica particles with higher aspect ratio were internalized quicker and more than those with low aspect ratio [125,149]. A few studies have investigated the effect of nanoparticle shape not only on uptake efficiency but also on the endocytic mechanisms involved in uptake [125,134,150]. Cylindric cationic poly(ethylene glycol) hydrogels with two different aspect ratios (1 and 3) were both taken up by HeLa cells by a combination of clathrin-mediated and caveolae-mediated endocytosis (based on cholesterol and tyrosine kinase dependence) [125]. On the other hand, in another study, the entry of cylindric, worm-like, and spherical silica particles in A549 and RAW264.7 cells was independent of cholesterol. Uptake of spherical silica particles was mainly clathrin-dependent, while internalization of worm-like and cylindric silica was primarily microtubule-dependent [134].

Similar studies on the effect of shape on the mechanism of internalization are challenging because of different complicating factors. Firstly, changing the shape also affects the dimensions of the nanomaterial. This means that to compare uptake of differently shaped objects, either the volume, maximum diameter, or a combination of the dimensions should be kept constant. Secondly, non-spherical objects can interact with the cell membrane with different orientation. Thus, depending on the orientation when interacting with the cell membrane, the contact area between the nano-sized object and the cell surface differs. It is thought that in these cases different mechanisms are then triggered based on the orientation of the nanoparticles [134,150]. This might, at least in part, explain why multiple pathways seem to be involved in the uptake of non-spherical nanomaterials.

**Nanoparticle rigidity:** Recently, there is a high interest in the effect of the rigidity of nanoparticles on the interactions at cellular level [109–110]. Simulations showed a higher energy barrier for the internalization of soft and easily deformable nanoparticles than for that of rigid particles [151–152]. However, nanoparticle–cell interactions cannot be described solely by the energy barrier required to bend the lipid membrane. Other biological factors are also involved, possibly explaining the contrasting results on the effect of rigidity on uptake [110,151–154]. Indeed, while some studies found higher uptake for the more rigid particles [151,153,155], another study suggested that softer particles enter in higher numbers [154]. In this latter study, it was also shown that the more rigid nanomaterial (Young’s modulus above 13.8 MPa) was internalized by cells at least in part via clathrin-mediated endocytosis, as opposed to the softer material [154]. Similarly, in another study, lipid covered PGLA particles with different Young’s modulus values in the range of gigapascals were also partially internalized by clathrin-mediated endocytosis [151].

**Understanding how nanoparticle properties affect the mechanism of uptake by cells:** Overall, the examples presented show that the effect of tunable nanoparticle parameters such as size, charge, and shape on the mechanisms of uptake by cells is often ambiguous. To further illustrate this complexity, Table 1 summarizes the few preliminary observations, which we discussed in this section, including references, which support and contrast them. We emphasize that Table 1 is far from complete and the observations included have been selected solely as an example to illustrate the complexity of often contrasting outcomes reported in literature.

**Table 1:** The table summarizes few selected examples that we discuss in Section 2.2 of observations reported in literature on the effect of nanoparticle properties on the mechanism of uptake by cells. References to literature with supporting as well as opposing observations are included (also discussed in Section 2.2). We emphasize that the table is far from complete and includes only a few examples, selected solely to illustrate the complexity of outcomes. In fact, we consider the observations listed still as preliminary, as also illustrated by apparently contrasting results in the opposing studies included (in most cases performed using different conditions and systems).

reported observation or preliminary statement	supporting studies	opposing studies

nanoparticles uptake efficiency decreases with increasing size	[107–109]	[112]
nanoparticles up to 100 nm in diameter enter through clathrin-dependent endocytosis	[107,113]	[114–116]
non-phagocytic cells can only internalize materials up to 200 nm	[8]	[107,114]
positively charged nanoparticles are internalized more efficiently than negatively charged or neutral nanoparticles	[114,124–125,128,130]	[126–127,129]
positively charged nanoparticles enter (at least partially) through clathrin-dependent endocytosis	[21,128,130]	[129]
nano-sized objects with small aspect ratios are internalized more efficiently	[112,133–134]	[114,136]
more rigid nanoparticles enter more efficiently than softer nanoparticles	[139,141,143]	[142]

A reason of this ambiguity and complexity might be that multiple mechanisms are triggered at the same time, as suggested by several studies [18,115,132,156–159]. It could also be that, besides the generally studied classical routes such as clathrin- and caveolae-mediated endocytosis, other less well-known pathways of internalization are involved in the uptake of nanomedicines, such as those briefly described in Section 2.1. Recently, computer simulations and in vitro studies of nanoparticle–membrane interactions have shown that the surface of nanomaterials can in itself induce several changes at the plasma membrane, by determining sol–gel transitions in the lipid bilayer and impairing lipid lateral diffusion [160–161], or by inducing bending of the plasma membrane [162–163], as already observed with certain viruses [164]. These changes in membrane dynamics might as well be a trigger for the endocytosis of nanoparticles via alternative mechanisms, which are not yet fully characterized.

Extracting conclusions from the available literature is additionally complicated by the fact that most studies have used different conditions with respect to many factors such as, for instance, the presence, source, and concentration of serum, but also the nanomaterial used, the cell type, and the methods applied to characterize the pathways involved. These differences clearly lead to different outcomes and apparently conflicting results. Only in a few cases, systematic studies using a series of nanomaterials of well-defined properties have been performed in order to try to disentangle the effect of multiple nanomaterial properties on the cellular uptake and on other biological effects [145,165]. Unfortunately, still no clear predictions can be made on how certain nanoparticle properties affect uptake efficiency and the mechanisms involved, and more work along these lines will be required [145,165]. Recent debates in the nanomedicine field pushed the community to address the issue of reproducibility and the development of standardized procedure in nanomedicine testing and application [166–167]. Similar efforts may help to reach a better understanding of how nanomaterial properties affect the mechanism of uptake by cells.

### 3 Intracellular fate

Another important aspect to consider for nanomedicine applications is the final fate of nano-sized materials following internalization. A recent review has discussed this aspect in more detail [85]. Regardless of the route of entry, many studies report that most nano-sized materials travel via the endosomes until reaching their final localization in the cell, which in most cases has been shown to be the lysosomal compartment [168–169]. In the lysosomes, nano-sized objects, including nanomedicines, may be degraded and release their content, if biodegradable, or may accumulate and persist [122]. While this can be very useful when the target is the lysosomes, it is well known that lysosomal accumulation constitutes an ulterior barrier for the delivery of drugs to any other intracellular target [85–86]. This has led to the development of strategies to induce escape from the endosomal compartment, including – among others – via the so-called “proton sponge effect” [170–171]. Materials capable to induce this proton sponge effect started to find their application in vivo only recently [172]. Several other strategies are being developed, including some inspired by viruses and bacteria capable to travel to other intracellular locations [173–176]. Several reviews fully focused on endo-lysosomal escape have summarised current efforts in this direction [174–177].

Another open question that is debated in the field is whether nano-sized materials, including nanomedicines, can end up in compartments other than the lysosomes. For example, in several reports it appears that nanoparticles can be transcytosed across endothelial cells [178–179]. However, the existence of a dedicated pathway, such as caveolae-mediated endocytosis, for transcytosis of macromolecules is still debated in the endocytosis community [96,102–104].

Understanding how cells recognize and internalize nano-sized materials can help us to address also questions regarding the intracellular fate of nano-sized materials and to define strategies to direct nanomedicines towards different intracellular locations or to promote drug release in cells.

### 4 Challenges in studying endocytosis of nano-sized materials in vitro

While studying the interactions between nanomaterials and cells is extremely challenging to perform in vivo, in vitro studies can help to unravel the mechanisms involved in their uptake. For such studies, the nanoparticle dispersion, the cell culture conditions, the cell line investigated, and the methods used to characterize the uptake mechanisms are all crucial. Unfortunately, there are often no agreements on how to perform uptake studies in a standardized way. Recently, this problem has gotten much attention in the nanomedicine field [166–167,180]. Within the nanosafety community, dedicated to the study of potential hazards of nanotechnologies, several efforts have been focused on the establishment of standardized procedures for nanomaterial handling and for cell interaction studies in order to ensure quality in nanosafety testing [181–183]. Some of the knowledge gained there could be helpful also in developing similar standards for studies aimed at characterizing how nano-sized objects, including nanomedicines, enter cells. In the following sections, we will describe some technical challenges concerning in vitro studies of the endocytosis of nano-sized materials.

#### 4.1 Nanoparticle dispersion in biological media: agglomeration and corona formation

One of the most important aspects to consider when studying nanoparticle interactions with cells, as well as when characterizing the mechanism for their internalization, is the stability over time and the potential agglomeration of nanoparticles in biological media. In fact, agglomeration can strongly affect the corona composition, the interaction with cells, as well as the pathways of internalization [59,184–185]. Thus, it is important to characterize the nanoparticle dispersion in the biological media in which the nanomedicine will be applied, and to monitor potential agglomeration and stability over time.

Additionally, studies in which nanoparticles are incubated on cells without serum or other biological fluids may lead to conclusions that are not relevant for biological applications and in vivo studies, because they do not take into account corona-related effects [186–189]. Given the impact of the corona on both recognition and internalization of nano-sized materials, it is important not only to include some biological fluid to allow corona formation, but also to define the appropriate conditions for each application [18,62–65,68–69]. For instance, there are many in vitro studies in which nanoparticles are tested on human cell lines using culture media containing a low percentage of foetal bovine serum [132–133,190–193]. Not only the percentage of serum, but also the species from which the serum originates, as well the use of serum heat inactivation, or the choice of anticoagulant used to prepare plasma are some of the factors affecting corona formation and potentially also the subsequent mechanisms of internalization [18,62–65,68–69]. Similar considerations should be made when characterizing the uptake of nanomedicines administered via other routes. In these cases biological fluids other than serum should be used [185,194].

On a more complex level, it is also known that the corona composition evolves over time [195–197]. Recent studies from Chan and colleagues are trying to explore how the corona composition affects the biological interactions of nanoparticles by performing mass spectrometry screenings and by developing computational models to predict the evolution of their protein corona [60–61,198]. Other studies are trying to understand not only whether certain biomolecules are present on the nanoparticle surface, but also their orientation, which might influence their recognition by cell receptors [17,199]. In order to take into account corona-related effects on nanoparticle interactions with cells, a precise workflow to characterize the corona composition has been proposed, which might help to compare different studies and to find a correlation between corona composition, serum composition and, ultimately, uptake mechanisms [41].

#### 4.2 Cell models

The cell type investigated and its tissue organization are other important factors that can affect the uptake mechanisms of nanoparticles. For example, not all pathways are active in all cell types: HepG2 cells have no endogenous caveolin-1, and therefore they are unable to internalize nanoparticles by caveolae-mediated endocytosis [200]. Similarly, many in vitro studies use immortalized or cancer cell lines, such as HEK293 or HeLa cells, which are easy to transfect and culture. However, these cells can behave quite differently in comparison to primary cells or cells isolated directly from tissues of patients. While, on one hand, the use of primary cells can be recommended, on the other hand, it is also a well-known obstacle for a detailed study of cellular pathways. Most primary cells are difficult to transfect and require interaction with other cell types for their proper differentiation [201–203]. Another important factor known to affect uptake pathways by cells in vitro is the differentiation of cells into barriers and the resulting cell polarization [159]. Uptake by cells grown to a polarized cell barrier is, in fact, different than uptake by the same cells when grown isolated or simply confluent [22,159].

Apart from simpler cell cultures and cell barriers, many advances have been made in the development of cellular models that can better reflect the more complex organization of cells in vivo. Models such as organoids or spheroids, which use one or multiple cell lines organized into 3D structures, have been developed and are likely to become useful also for nanomedicine uptake studies.

#### 4.3 Methods to characterize uptake mechanisms

Further difficulties in the study of the uptake mechanisms of nano-sized materials, such as nanomedicines, arise from the fact that endocytosis represents a complex cellular process with many molecules, feedback loops, and signalling cascades involved. The endocytosis field is still very active and constantly progressing [71,77,204]. Many processes and molecular details of these pathways are still unknown. For instance, in recent years much attention has been paid to non-canonical pathways of endocytosis [71,91–92], which are often more difficult to study, but which might as well constitute possible routes for the internalization of nano-sized materials. In light of this complexity, the tendency within the nanomedicine field to classify the pathways of internalization of drug carriers as merely macropinocytosis, clathrin-dependent, or caveolae-dependent is for sure an oversimplification. Furthermore, the classification and description of the different mechanisms of uptake by cells are often revised and corrected in the endocytosis field, as research progresses. This is an example of the challenges that interdisciplinary fields such as nanomedicine need to face. In this context, a closer connection with the cell biology and endocytosis communities is desirable [113].

Typical experiments to characterize uptake mechanisms are carried out by altering cellular pathways using different methods in order to determine their involvement in nanoparticle uptake. However, it is well established that perturbation of a cellular mechanism might as well lead to the alteration of other mechanisms. Therefore, when performing such studies, it is important to have appropriate controls to verify the effect of the selected treatment on the pathway of interest and exclude potential artefacts of this kind [22,133,158].

The selection of appropriate controls poses further challenges. For example, fluorescently labelled low-density lipoprotein and transferrin can be used as markers for clathrin-mediated endocytosis [205–206], dextran as a fluid phase marker for phagocytosis and for the CLEE/GEEC pathway [193], and LacCer (C5-lactosylceramide) for cholesterol-dependent uptake [133,207]. However, while cholera toxin and SV40 were previously used as markers of caveolae-mediated endocytosis [95], they have been found to enter cells using preferentially other routes and thus should not be used anymore as markers for this pathway [208–210]. Furthermore, selecting an appropriate control marker can be challenging for several of the more recently described clathrin- and caveolae- independent pathways.

Next to the difficulties related to the choice of control markers, the fact that the endocytic pathways are strongly interconnected and that some components take part in multiple mechanisms also complicates further the interpretation of results. As an example of this, cytochalasin D, an inhibitor of actin polymerization [211], has often been used to test the involvement of macropinocytosis and phagocytosis in the uptake of nanomaterials. However, actin has been shown to be important also for other mechanisms, including clathrin-mediated endocytosis and caveolae-mediated endocytosis. Hence, perturbing the activity of actin affects multiple pathways at the same time [212].

Several techniques can be used for studying the endocytic mechanisms of nano-sized materials, each one with its advantages and drawbacks [22,133]. Among those, RNA interference (RNAi) is often used to shut down the expression of a single protein or even a single isoform. However, the complete depletion of the protein of interest after RNAi requires at least 48–96 h and during this time cells can adapt, for example by upregulating other proteins of the same family or other pathways. Moreover, RNAi does not guarantee the total depletion of a protein from the cell, and in some cases the partial reduction of a protein is not sufficient to fully inhibit its function [22].

Apart from RNAi, so far, many studies on the uptake of nano-sized materials make use of transport inhibitors, whose action on cells is instead very fast. However, some of these inhibitors lack specificity, they might interfere with multiple pathways, and they can cause cellular toxicity [22,133,158,213]. In contrast to these approaches to block uptake pathways, other strategies can be found in which proteins are instead overexpressed, in order to eventually detect an increase in nanoparticle uptake [17]. Nevertheless, also these methods might lead to artefacts, since the overexpression might induce the activation of a pathway that may not be active under physiological conditions [22,214]. Furthermore, some proteins act as heterodimers or in concert with other molecular partners, thus their overexpression might not produce any detectable effect if not combined with the overexpression of those partners as well. Overexpression is often used to allow for the visualization of fluorescently tagged molecules by microscopy. However, also this might lead to additional artefacts. For example, it has been shown that overexpression of GFP-tagged caveolin 1 (CAV1-GFP), the main constituent of caveolae-mediated endocytosis, leads to the creation of artefacts, such as the observation of a specialized endosomal compartment for caveolae, the “caveosome” [214].

Recent advances in cellular imaging and gene editing could overcome some of these issues. For instance, the use of stably transfected cell lines might be a good solution when the total depletion of a protein is required to shut down a pathway (often difficult to achieve with techniques such as RNAi), but also for expressing labelled proteins at physiological levels, thereby avoiding the aforementioned artefacts connected to overexpression. The creation of specific transgenic cell lines to study nanoparticle uptake has been greatly enhanced by gene editing techniques such as CRISPR/CAS9, which allows to cut genes in a much more specific and efficient way than with previously existing methods [215–216]. Similar methodologies can improve our understanding of the involvement of specific proteins in the internalization of nanoparticles.

Other recently developed techniques that are available in cell biology to characterize pathways and that have not yet been used to study the uptake of nano-sized materials may provide novel insights into this difficult question. For instance, so-called OMICS approaches based on large-scale proteomics and full genome screenings could be of particular use [217–219]. While most of the “classical” methods mentioned so far require previous knowledge on the mechanisms of uptake by cells, a reverse approach could allow for discovering novel targets not yet associated with the endocytosis of nanomaterials.

Overall, it is clear that none of these different methods, alone, can provide a full picture of the mechanisms that nano-sized materials use to interact with cells since they all display different advantages and pitfalls [22,133,158]. The combination of different techniques and the application of proper controls could help us to gain a better knowledge of the endocytic processes involved in the uptake of nano-sized materials.

## Conclusion

Nanomedicine aims at delivering drugs more efficiently to their target to treat diseases. Designing the properties of nano-sized materials to be able to control the interactions and the behaviour at cell level is one of the key steps required for successful targeting. Nanomedicines can be functionalized by the addition of targeting moieties to be recognized by specific receptors on the targeted cells. However, achieving this initial recognition for active targeting still holds many challenges.

Additionally, it has emerged that when nano-sized materials are applied in a biological environment, corona formation affects the initial recognition by cells, as well as the following mechanisms of internalization. However, many aspects of the initial recognition of nano-sized materials by cell receptors still need to be understood. Similarly, how the initial recognition affects the following mechanisms of internalization remains to be elucidated and an agreement in the scientific community about the pathways that nano-sized materials, such as nanomedicines, use to enter cells is still missing.

Several factors complicate these studies and make it difficult to draw clear conclusions. The field of endocytosis is still very active and novel pathways are still being described. At the same time there are limits and challenges regarding the many different methods available to characterize uptake mechanisms and the lack of standardized procedures makes it difficult to draw conclusions from available studies. Using a combination of methods and appropriate controls to study the mechanisms by which cells internalize nano-sized materials could potentially help us to understand how these are affected by nanomaterial properties. In this way, nanomedicine design could be tuned to achieve the desired outcomes at cell level and engineer nanomaterials for more efficient drug targeting.

## References

[1] Wolfram J, Ferrari M (2019). Nano Today.

[2] Peer D, Karp J M, Hong S, Farokhzad O C, Margalit R, Langer R (2007). Nat Nanotechnol.

[3] Shi J, Kantoff P W, Wooster R, Farokhzad O C (2017). Nat Rev Cancer.

[4] Sandvig K, van Deurs B (2005). Gene Ther.

[5] Bareford L M, Swaan P W (2007). Adv Drug Delivery Rev.

[6] Bobo D, Robinson K J, Islam J, Thurecht K J, Corrie S R (2016). Pharm Res.

[7] Petros R A, DeSimone J M (2010). Nat Rev Drug Discovery.

[8] Duan X, Li Y (2013). Small.

[9] Chauhan V P, Jain R K (2013). Nat Mater.

[10] Allen T M (2002). Nat Rev Cancer.

[11] Villaverde G, Baeza A (2019). Beilstein J Nanotechnol.

[12] Bertrand N, Wu J, Xu X, Kamaly N, Farokhzad O C (2014). Adv Drug Delivery Rev.

[13] Danhier F, Feron O, Préat V (2010). J Controlled Release.

[14] Sykes E A, Chen J, Zheng G, Chan W C W (2014). ACS Nano.

[15] Salvati A, Pitek A S, Monopoli M P, Prapainop K, Bombelli F B, Hristov D R, Kelly P M, Åberg C, Mahon E, Dawson K A (2013). Nat Nanotechnol.

[16] Hadjidemetriou M, Al-Ahmady Z, Mazza M, Collins R F, Dawson K, Kostarelos K (2015). ACS Nano.

[17] Lara S, Alnasser F, Polo E, Garry D, Lo Giudice M C, Hristov D R, Rocks L, Salvati A, Yan Y, Dawson K A (2017). ACS Nano.

[18] Francia V, Yang K, Deville S, Reker-Smit C, Nelissen I, Salvati A (2019). ACS Nano.

[19] Duncan R, Richardson S C W (2012). Mol Pharmaceutics.

[20] Oh N, Park J H (2014). Int J Nanomed.

[21] Sahay G, Alakhova D Y, Kabanov A V (2010). J Controlled Release.

[22] Iversen T-G, Skotland T, Sandvig K (2011). Nano Today.

[23] Sudimack J, Lee R J (2000). Adv Drug Delivery Rev.

[24] Daniels T R, Bernabeu E, Rodríguez J A, Patel S, Kozman M, Chiappetta D A, Holler E, Ljubimova J Y, Helguera G, Penichet M L (2012). Biochim Biophys Acta, Gen Subj.

[25] Mattheolabakis G, Milane L, Singh A, Amiji M M (2015). J Drug Targeting.

[26] Wilhelm S, Tavares A J, Dai Q, Ohta S, Audet J, Dvorak H F, Chan W C W (2016). Nat Rev Mater.

[27] McNeil S E (2016). Nat Rev Mater.

[28] Lammers T, Kiessling F, Ashford M, Hennink W, Crommelin D, Storm G (2016). Nat Rev Mater.

[29] Danhier F (2016). J Controlled Release.

[30] Olivier V, Meisen I, Meckelein B, Hirst T R, Peter-Katalinic J, Schmidt M A, Frey A (2003). Bioconjugate Chem.

[31] Aubin-Tam M-E, Hwang W, Hamad-Schifferli K (2009). Proc Natl Acad Sci U S A.

[32] Kogot J M, Parker A M, Lee J, Blaber M, Strouse G F, Logan T M (2009). Bioconjugate Chem.

[33] Muro S (2012). J Controlled Release.

[34] Mahon E, Salvati A, Baldelli Bombelli F, Lynch I, Dawson K A (2012). J Controlled Release.

[35] Ruoslahti E, Bhatia S N, Sailor M J (2010). J Cell Biol.

[36] Monopoli M P, Åberg C, Salvati A, Dawson K A (2012). Nat Nanotechnol.

[37] Mahmoudi M, Lynch I, Ejtehadi M R, Monopoli M P, Bombelli F B, Laurent S (2011). Chem Rev.

[38] Cedervall T, Lynch I, Lindman S, Berggard T, Thulin E, Nilsson H, Dawson K A, Linse S (2007). Proc Natl Acad Sci U S A.

[39] Schöttler S, Landfester K, Mailänder V (2016). Angew Chem, Int Ed.

[40] Tonigold M, Simon J, Estupiñán D, Kokkinopoulou M, Reinholz J, Kintzel U, Kaltbeitzel A, Renz P, Domogalla M P, Steinbrink K (2018). Nat Nanotechnol.

[41] Chetwynd A J, Wheeler K E, Lynch I (2019). Nano Today.

[42] Harris J M, Martin N E, Modi M (2001). Clin Pharmacokinet.

[43] Howard M D, Jay M, Dziubla T D, Lu X (2008). J Biomed Nanotechnol.

[44] García K P, Zarschler K, Barbaro L, Barreto J A, O'Malley W, Spiccia L, Stephan H, Graham B (2014). Small.

[45] Safavi-Sohi R, Maghari S, Raoufi M, Jalali S A, Hajipour M J, Ghassempour A, Mahmoudi M (2016). ACS Appl Mater Interfaces.

[46] Schöttler S, Becker G, Winzen S, Steinbach T, Mohr K, Landfester K, Mailänder V, Wurm F R (2016). Nat Nanotechnol.

[47] Dai Q, Walkey C, Chan W C W (2014). Angew Chem, Int Ed.

[48] Lundqvist M, Stigler J, Elia G, Lynch I, Cedervall T, Dawson K A (2008). Proc Natl Acad Sci U S A.

[49] Caracciolo G, Cardarelli F, Pozzi D, Salomone F, Maccari G, Bardi G, Capriotti A L, Cavaliere C, Papi M, Laganà A (2013). ACS Appl Mater Interfaces.

[50] Deng Z J, Liang M, Monteiro M, Toth I, Minchin R F (2011). Nat Nanotechnol.

[51] Gilleron J, Querbes W, Zeigerer A, Borodovsky A, Marsico G, Schubert U, Manygoats K, Seifert S, Andree C, Stöter M (2013). Nat Biotechnol.

[52] Akinc A, Querbes W, De S, Qin J, Frank-Kamenetsky M, Jayaprakash K N, Jayaraman M, Rajeev K G, Cantley W L, Dorkin J R (2010). Mol Ther.

[53] Akinc A, Maier M A, Manoharan M, Fitzgerald K, Jayaraman M, Barros S, Ansell S, Du X, Hope M J, Madden T D (2019). Nat Nanotechnol.

[54] Tenzer S, Docter D, Rosfa S, Wlodarski A, Kuharev J, Rekik A, Knauer S K, Bantz C, Nawroth T, Bier C (2011). ACS Nano.

[55] Fleischer C C, Payne C K (2014). J Phys Chem B.

[56] Zhang Z, Wang C, Zha Y, Hu W, Gao Z, Zang Y, Chen J, Zhang J, Dong L (2015). ACS Nano.

[57] Prapainop K, Witter D P, Wentworth P (2012). J Am Chem Soc.

[58] Gaspar R (2013). Nat Nanotechnol.

[59] Nel A E, Mädler L, Velegol D, Xia T, Hoek E M V, Somasundaran P, Klaessig F, Castranova V, Thompson M (2009). Nat Mater.

[60] Lazarovits J, Sindhwani S, Tavares A J, Zhang Y, Song F, Audet J, Krieger J R, Syed A M, Stordy B, Chan W C W (2019). ACS Nano.

[61] Walkey C D, Olsen J B, Song F, Liu R, Guo H, Olsen D W H, Cohen Y, Emili A, Chan W C W (2014). ACS Nano.

[62] Pisani C, Rascol E, Dorandeu C, Gaillard J-C, Charnay C, Guari Y, Chopineau J, Armengaud J, Devoisselle J-M, Prat O (2017). PLoS One.

[63] Schöttler S, Klein K, Landfester K, Mailänder V (2016). Nanoscale.

[64] Mirshafiee V, Kim R, Mahmoudi M, Kraft M L (2016). Int J Biochem Cell Biol.

[65] Lesniak A, Campbell A, Monopoli M P, Lynch I, Salvati A, Dawson K A (2010). Biomaterials.

[66] Monopoli M P, Walczyk D, Campbell A, Elia G, Lynch I, Baldelli Bombelli F, Dawson K A (2011). J Am Chem Soc.

[67] Partikel K, Korte R, Mulac D, Humpf H-U, Langer K (2019). Beilstein J Nanotechnol.

[68] Hajipour M J, Laurent S, Aghaie A, Rezaee F, Mahmoudi M (2014). Biomater Sci.

[69] Corbo C, Molinaro R, Tabatabaei M, Farokhzad O C, Mahmoudi M (2017). Biomater Sci.

[70] Doherty G J, McMahon H T (2009). Annu Rev Biochem.

[71] Ferreira A P A, Boucrot E (2018). Trends Cell Biol.

[72] Johannes L, Mayor S (2010). Cell.

[73] Johannes L, Parton R G, Bassereau P, Mayor S (2015). Nat Rev Mol Cell Biol.

[74] Graham T R, Kozlov M M (2010). Curr Opin Cell Biol.

[75] Posor Y, Eichhorn-Grünig M, Haucke V (2015). Biochim Biophys Acta, Mol Cell Biol Lipids.

[76] Johannes L, Wunder C, Shafaq-Zadah M (2016). J Mol Biol.

[77] McMahon H T, Boucrot E (2015). J Cell Sci.

[78] McMahon H T, Gallop J L (2005). Nature.

[79] Boucrot E, Pick A, Çamdere G, Liska N, Evergren E, McMahon H T, Kozlov M M (2012). Cell.

[80] Simunovic M, Manneville J-B, Renard H-F, Evergren E, Raghunathan K, Bhatia D, Kenworthy A K, Voth G A, Prost J, McMahon H T (2017). Cell.

[81] Römer W, Pontani L-L, Sorre B, Rentero C, Berland L, Chambon V, Lamaze C, Bassereau P, Sykes C, Gaus K (2010). Cell.

[82] Bashkirov P V, Akimov S A, Evseev A I, Schmid S L, Zimmerberg J, Frolov V A (2008). Cell.

[83] Pucadyil T J, Schmid S L (2008). Cell.

[84] Johannes L, Wunder C, Bassereau P (2014). Cold Spring Harbor Perspect Biol.

[85] Patel S, Kim J, Herrera M, Mukherjee A, Kabanov A V, Sahay G (2019). Adv Drug Delivery Rev.

[86] Canton I, Battaglia G (2012). Chem Soc Rev.

[87] Fujimoto L M, Roth R, Heuser J E, Schmid S L (2000). Traffic.

[88] Kaksonen M, Toret C P, Drubin D G (2006). Nat Rev Mol Cell Biol.

[89] Merrifield C J, Perrais D, Zenisek D (2005). Cell.

[90] Cureton D K, Massol R H, Whelan S P J, Kirchhausen T (2010). PLoS Pathog.

[91] Hansen C G, Nichols B J (2009). J Cell Sci.

[92] Sandvig K, Pust S, Skotland T, van Deurs B (2011). Curr Opin Cell Biol.

[93] Gao Y-s, Hubbert C C, Lu J, Lee Y-S, Lee J-Y, Yao T-P (2007). Mol Cell Biol.

[94] Grimmer S, van Deurs B, Sandvig K (2002). J Cell Sci.

[95] Pelkmans L, Helenius A (2002). Traffic.

[96] Kirkham M, Parton R G (2005). Biochim Biophys Acta, Mol Cell Res.

[97] Chaudhary N, Gomez G A, Howes M T, Lo H P, McMahon K-A, Rae J A, Schieber N L, Hill M M, Gaus K, Yap A S (2014). PLoS Biol.

[98] Stoeber M, Stoeck I K, Hänni C, Bleck C K E, Balistreri G, Helenius A (2012). EMBO J.

[99] Boucrot E, Howes M T, Kirchhausen T, Parton R G (2011). J Cell Sci.

[100] Oh P, Borgström P, Witkiewicz H, Li Y, Borgström B J, Chrastina A, Iwata K, Zinn K R, Baldwin R, Testa J E (2007). Nat Biotechnol.

[101] van Deurs B, Roepstorff K, Hommelgaard A M, Sandvig K (2003). Trends Cell Biol.

[102] Shvets E, Bitsikas V, Howard G, Hansen C G, Nichols B J (2015). Nat Commun.

[103] Thomsen P, Roepstorff K, Stahlhut M, van Deurs B (2002). Mol Biol Cell.

[104] Pelkmans L, Zerial M (2005). Nature.

[105] Gordon S (2016). Immunity.

[106] Lim J P, Gleeson P A (2011). Immunol Cell Biol.

[107] Venkataraman S, Hedrick J L, Ong Z Y, Yang C, Ee P L R, Hammond P T, Yang Y Y (2011). Adv Drug Delivery Rev.

[108] Lorenz S, Hauser C P, Autenrieth B, Weiss C K, Landfester K, Mailänder V (2010). Macromol Biosci.

[109] Yi X, Shi X, Gao H (2011). Phys Rev Lett.

[110] Anselmo A C, Mitragotri S (2016). Adv Drug Delivery Rev.

[111] Niu Y, Yu M, Meka A, Liu Y, Zhang J, Yang Y, Yu C (2016). J Mater Chem B.

[112] Kelf T A, Sreenivasan V K A, Sun J, Kim E J, Goldys E M, Zvyagin A V (2010). Nanotechnology.

[113] Editorial 'Time to deliver' (2014). Nat Biotechnol.

[114] Akinc A, Battaglia G (2013). Cold Spring Harbor Perspect Biol.

[115] Rejman J, Oberle V, Zuhorn I S, Hoekstra D (2004). Biochem J.

[116] Lerch S, Dass M, Musyanovych A, Landfester K, Mailänder V (2013). Eur J Pharm Biopharm.

[117] Foged C, Brodin B, Frokjaer S, Sundblad A (2005). Int J Pharm.

[118] Decuzzi P, Ferrari M (2007). Biomaterials.

[119] Gao H, Shi W, Freund L B (2005). Proc Natl Acad Sci U S A.

[120] Chithrani B D, Ghazani A A, Chan W C W (2006). Nano Lett.

[121] Zauner W, Farrow N A, Haines A M R (2001). J Controlled Release.

[122] Shapero K, Fenaroli F, Lynch I, Cottell D C, Salvati A, Dawson K A (2011). Mol BioSyst.

[123] Wang S-H, Lee C-W, Chiou A, Wei P-K (2010). J Nanobiotechnol.

[124] Blanco E, Shen H, Ferrari M (2015). Nat Biotechnol.

[125] Gratton S E A, Ropp P A, Pohlhaus P D, Luft J C, Madden V J, Napier M E, DeSimone J M (2008). Proc Natl Acad Sci U S A.

[126] Lai S K, Hida K, Man S T, Chen C, Machamer C, Schroer T A, Hanes J (2007). Biomaterials.

[127] Agarwal R, Singh V, Jurney P, Shi L, Sreenivasan S V, Roy K (2013). Proc Natl Acad Sci U S A.

[128] Kirchhausen T, Owen D, Harrison S C (2014). Cold Spring Harbor Perspect Biol.

[129] Fotin A, Cheng Y, Sliz P, Grigorieff N, Harrison S C, Kirchhausen T, Walz T (2004). Nature.

[130] Saffarian S, Cocucci E, Kirchhausen T (2009). PLoS Biol.

[131] dos Santos T, Varela J, Lynch I, Salvati A, Dawson K A (2011). Small.

[132] dos Santos T, Varela J, Lynch I, Salvati A, Dawson K A (2011). PLoS One.

[133] Vercauteren D, Vandenbroucke R E, Jones A T, Rejman J, Demeester J, De Smedt S C, Sanders N N, Braeckmans K (2010). Mol Ther.

[134] Herd H, Daum N, Jones A T, Huwer H, Ghandehari H, Lehr C-M (2013). ACS Nano.

[135] Gessner A, Lieske A, Paulke B R, Müller R H (2002). Eur J Pharm Biopharm.

[136] Fröhlich E (2012). Int J Nanomed.

[137] Hühn D, Kantner K, Geidel C, Brandholt S, De Cock I, Soenen S J H, Rivera_Gil P, Montenegro J-M, Braeckmans K, Müllen K (2013). ACS Nano.

[138] Chen L, Mccrate J M, Lee J C-M, Li H (2011). Nanotechnology.

[139] Schrade A, Mailänder V, Ritz S, Landfester K, Ziener U (2012). Macromol Biosci.

[140] Xiao K, Li Y, Luo J, Lee J S, Xiao W, Gonik A M, Agarwal R G, Lam K S (2011). Biomaterials.

[141] Bannunah A M, Vllasaliu D, Lord J, Stolnik S (2014). Mol Pharmaceutics.

[142] Dausend J, Musyanovych A, Dass M, Walther P, Schrezenmeier H, Landfester K, Mailänder V (2008). Macromol Biosci.

[143] Harush-Frenkel O, Rozentur E, Benita S, Altschuler Y (2008). Biomacromolecules.

[144] Li Y, Kröger M, Liu W K (2015). Nanoscale.

[145] Carnovale C, Bryant G, Shukla R, Bansal V (2019). ACS Omega.

[146] Qiu Y, Liu Y, Wang L, Xu L, Bai R, Ji Y, Wu X, Zhao Y, Li Y, Chen C (2010). Biomaterials.

[147] Chithrani B D, Chan W C W (2007). Nano Lett.

[148] Dasgupta S, Auth T, Gompper G (2014). Nano Lett.

[149] Huang X, Teng X, Chen D, Tang F, He J (2010). Biomaterials.

[150] Kinnear C, Moore T L, Rodriguez-Lorenzo L, Rothen-Rutishauser B, Petri-Fink A (2017). Chem Rev.

[151] Sun J, Zhang L, Wang J, Feng Q, Liu D, Yin Q, Xu D, Wei Y, Ding B, Shi X (2015). Adv Mater (Weinheim, Ger).

[152] Shen Z, Ye H, Yi X, Li Y (2019). ACS Nano.

[153] Palomba R, Palange A L, Rizzuti I F, Ferreira M, Cervadoro A, Barbato M G, Canale C, Decuzzi P (2018). ACS Nano.

[154] Guo P, Liu D, Subramanyam K, Wang B, Yang J, Huang J, Auguste D T, Moses M A (2018). Nat Commun.

[155] Anselmo A C, Zhang M, Kumar S, Vogus D R, Menegatti S, Helgeson M E, Mitragotri S (2015). ACS Nano.

[156] Kuhn D A, Vanhecke D, Michen B, Blank F, Gehr P, Petri-Fink A, Rothen-Rutishauser B (2014). Beilstein J Nanotechnol.

[157] O’ Neill M J, Guo J, Byrne C, Darcy R, O’ Driscoll C M (2011). Int J Pharm.

[158] Francia V, Reker-Smit C, Boel G, Salvati A (2019). Nanomedicine (London, U K).

[159] Francia V, Aliyandi A, Salvati A (2018). Nanoscale.

[160] Wang B, Zhang L, Bae S C, Granick S (2008). Proc Natl Acad Sci U S A.

[161] Rossi G, Barnoud J, Monticelli L (2014). J Phys Chem Lett.

[162] Zhao W, Hanson L, Lou H-Y, Akamatsu M, Chowdary P D, Santoro F, Marks J R, Grassart A, Drubin D G, Cui Y (2017). Nat Nanotechnol.

[163] Bahrami A H, Lipowsky R, Weikl T R (2016). Soft Matter.

[164] Ewers H, Römer W, Smith A E, Bacia K, Dmitrieff S, Chai W, Mancini R, Kartenbeck J, Chambon V, Berland L (2010). Nat Cell Biol.

[165] Xu M, Soliman M G, Sun X, Pelaz B, Feliu N, Parak W J, Liu S (2018). ACS Nano.

[166] Leong H S, Butler K S, Brinker C J, Azzawi M, Conlan S, Dufés C, Owen A, Rannard S, Scott C, Chen C (2019). Nat Nanotechnol.

[167] Lammers T, Storm G (2019). Nat Nanotechnol.

[168] Sahay G, Querbes W, Alabi C, Eltoukhy A, Sarkar S, Zurenko C, Karagiannis E, Love K, Chen D, Zoncu R (2013). Nat Biotechnol.

[169] Pangarkar C, Dinh A-T, Mitragotri S (2012). J Controlled Release.

[170] Behr J (1997). Chimia.

[171] Rehman Z u, Hoekstra D, Zuhorn I S (2013). ACS Nano.

[172] El-Sayed A, Futaki S, Harashima H (2009). AAPS J.

[173] Krpetić Ž, Saleemi S, Prior I A, Sée V, Qureshi R, Brust M (2011). ACS Nano.

[174] Chou L Y T, Ming K, Chan W C W (2011). Chem Soc Rev.

[175] Varkouhi A K, Scholte M, Storm G, Haisma H J (2011). J Controlled Release.

[176] Martens T F, Remaut K, Demeester J, De Smedt S C, Braeckmans K (2014). Nano Today.

[177] Smith S A, Selby L I, Johnston A P R, Such G K (2019). Bioconjugate Chem.

[178] Chai G-H, Hu F-Q, Sun J, Du Y-Z, You J, Yuan H (2014). Mol Pharmaceutics.

[179] Ghaffarian R, Bhowmick T, Muro S (2012). J Controlled Release.

[180] Rezaei G, Daghighi S M, Haririan I, Yousefi I, Raoufi M, Rezaee F, Dinarvand R (2019). Colloids Surf, B.

[181] Krug H F (2014). Angew Chem, Int Ed.

[182] Haase A, Lynch I (2018). NanoImpact.

[183] Editorial 'Join the, dialogue' (2012). Nat Nanotechnol.

[184] Malcolm D W, Varghese J J, Sorrells J E, Ovitt C E, Benoit D S W (2018). ACS Nano.

[185] Lazzari S, Moscatelli D, Codari F, Salmona M, Morbidelli M, Diomede L (2012). J Nanopart Res.

[186] Lesniak A, Fenaroli F, Monopoli M P, Åberg C, Dawson K A, Salvati A (2012). ACS Nano.

[187] Kim J A, Salvati A, Åberg C, Dawson K A (2014). Nanoscale.

[188] Mazzolini J, Weber R J M, Chen H-S, Khan A, Guggenheim E, Shaw R K, Chipman J K, Viant M R, Rappoport J Z (2016). Biol Bull (Woods Hole, MA, U S).

[189] Digiacomo L, Cardarelli F, Pozzi D, Palchetti S, Digman M A, Gratton E, Capriotti A L, Mahmoudi M, Caracciolo G (2017). Nanoscale.

[190] Voigt J, Christensen J, Shastri V P (2014). Proc Natl Acad Sci U S A.

[191] Hild W, Pollinger K, Caporale A, Cabrele C, Keller M, Pluym N, Buschauer A, Rachel R, Tessmar J, Breunig M (2010). Proc Natl Acad Sci U S A.

[192] Lunov O, Syrovets T, Loos C, Beil J, Delacher M, Tron K, Nienhaus G U, Musyanovych A, Mailänder V, Landfester K (2011). ACS Nano.

[193] Al Soraj M, He L, Peynshaert K, Cousaert J, Vercauteren D, Braeckmans K, De Smedt S C, Jones A T (2012). J Controlled Release.

[194] Choi H S, Ashitate Y, Lee J H, Kim S H, Matsui A, Insin N, Bawendi M G, Semmler-Behnke M, Frangioni J V, Tsuda A (2010). Nat Biotechnol.

[195] Barrán-Berdón A L, Pozzi D, Caracciolo G, Capriotti A L, Caruso G, Cavaliere C, Riccioli A, Palchetti S, Laganà A (2013). Langmuir.

[196] Casals E, Pfaller T, Duschl A, Oostingh G J, Puntes V (2010). ACS Nano.

[197] Lundqvist M, Stigler J, Cedervall T, Berggård T, Flanagan M B, Lynch I, Elia G, Dawson K (2011). ACS Nano.

[198] Liu R, Jiang W, Walkey C D, Chan W C W, Cohen Y (2015). Nanoscale.

[199] Kelly P M, Åberg C, Polo E, O’Connell A, Cookman J, Fallon J, Krpetić Ž, Dawson K A (2015). Nat Nanotechnol.

[200] Fujimoto T, Kogo H, Nomura R, Une T (2000). J Cell Sci.

[201] Schimpel C, Teubl B, Absenger M, Meindl C, Fröhlich E, Leitinger G, Zimmer A, Roblegg E (2014). Mol Pharmaceutics.

[202] Gamboa J M, Leong K W (2013). Adv Drug Delivery Rev.

[203] Costa E C, Gaspar V M, Marques J G, Coutinho P, Correia I J (2013). PLoS One.

[204] Mayor S, Parton R G, Donaldson J G (2014). Cold Spring Harbor Perspect Biol.

[205] Harding C, Heuser J, Stahl P (1983). J Cell Biol.

[206] Vasile E, Simionescu M, Simionescu N (1983). J Cell Biol.

[207] Marks D L, Singh R D, Choudhury A, Wheatley C L, Pagano R E (2005). Methods.

[208] Lajoie P, Kojic L D, Nim S, Li L, Dennis J W, Nabi I R (2009). J Cell Mol Med.

[209] Torgersen M L, Skretting G, van Deurs B, Sandvig K (2001). J Cell Sci.

[210] Damm E-M, Pelkmans L, Kartenbeck J, Mezzacasa A, Kurzchalia T, Helenius A (2005). J Cell Biol.

[211] Schliwa M (1982). J Cell Biol.

[212] Yarar D, Waterman-Storer C M, Schmid S L (2005). Mol Biol Cell.

[213] Ivanov A I, Ivanov A I (2008). Pharmacological Inhibition of Endocytic Pathways: Is It Specific Enough to Be Useful?. Exocytosis and Endocytosis.

[214] Hayer A, Stoeber M, Ritz D, Engel S, Meyer H H, Helenius A (2010). J Cell Biol.

[215] Roberts B, Haupt A, Tucker A, Grancharova T, Arakaki J, Fuqua M A, Nelson A, Hookway C, Ludmann S A, Mueller I A (2017). Mol Biol Cell.

[216] Khan A O, Simms V A, Pike J A, Thomas S G, Morgan N V (2017). Sci Rep.

[217] Wittrup A, Zhang S-H, Svensson K J, Kucharzewska P, Johansson M C, Morgelin M, Belting M (2010). Proc Natl Acad Sci U S A.

[218] Yau E H, Rana T M (2018). Methods Mol Biol (N Y, NY, U S).

[219] Gosney J A, Wilkey D W, Merchant M L, Ceresa B P (2018). J Biol Chem.

